# A differentiation program induced by bone morphogenetic proteins 4 and 7 in endodermal epithelial cells provides the molecular basis for efficient nutrient transport by the chicken yolk sac

**DOI:** 10.1002/dvdy.129

**Published:** 2019-11-12

**Authors:** Raimund Bauer, Philipp Tondl, Wolfgang J. Schneider

**Affiliations:** ^1^ Center for Pathobiochemistry and Genetics Institute of Medical Chemistry, Medical University of Vienna Vienna Austria; ^2^ Department of Chemistry University of Natural Resources and Life Sciences Vienna Austria; ^3^ Department of Medical Biochemistry Max Perutz Labs, Medical University of Vienna Vienna Austria

**Keywords:** area vasculosa, feto‐maternal interface, nutrient transfer, paracrine signaling, triglyceride‐rich lipoproteins

## Abstract

**Background:**

The mammalian yolk sac provides nutrients for the growing fetus during critical early developmental processes such as neural tube closure, which precedes the functional maturation of the placenta. In contrast, oviparous species such as the chicken rely solely on the yolk sac for transfer of nutrients from the yolk to the developing embryo. However, the molecular mechanisms that provide the yolk sac with nutrient transfer competence remain poorly understood.

**Results:**

We demonstrate that the chicken endodermal epithelial cells (EEC), which are in close contact with the yolk, gain their nutrient‐transport competence by a paracrine crosstalk with the blood‐vessel forming mesodermal cell layer. Bone morphogenetic proteins (BMP) 4 and 7 produced by ectodermal and mesodermal cell layers likely initiate a differentiation program of EECs during the transition from the area vitellina to the area vasculosa. BMPs, by inducing SMAD signaling, promote the up‐regulation of endocytic receptor expression and thereby provide the EECs with the molecular machinery to produce triglyceride‐rich lipoprotein particles.

**Conclusion:**

This paracrine signaling cascade may constitute the basis for the EEC‐mediated mechanism underlying the efficient uptake, degradation, resynthesis, and transfer of yolk‐derived nutrients into the embryonic circulation, which assures proper energy supply and development of the growing fetus.

## Key Findings

- Endodermal epithelial cells gain their nutrient transport competence during the transition from the area vitellina to the area vasculosa.
- BMPs signal from the ecto/mesodermal cell sheet to the underlying endoderm, thereby initiating endoderm specification.
- BMPs thereby provide the endoderm with nutrient transport competence which allows proper nutrition of the growing embryo.

## 1 INTRODUCTION

In amniotic vertebrates, the yolk sac is the first organ that mediates the transport of nutrients from maternal sources to the growing embryo.^1^, ^2^, ^3^ In mammals, the yolk sac is of major importance before the formation and functional maturation of the chorioallantoic placenta, a highly critical time in embryonic development during which early organogenesis and neural tube closure occur. In contrast, developing embryos of oviparous species such as the chicken depend exclusively on the yolk sac, as it represents the sole organ that transfers maternally derived nutrients stored in the yolk to the growing fetus. Nevertheless, structural and functional features of the yolk sac are highly conserved between species, and comparative RNA sequencing data revealed a significant conservation of the yolk sac transcriptome from human, murine, and chicken embryos.^1^, ^2^, ^3^, ^4^

In our previous study, we reported that the chicken yolk sac endodermal epithelial cells (EECs), which mediate the efficient uptake, degradation, and resynthesis of yolk nutrients, differentiate into a metabolically highly active type of cells as they become competent for the uptake of nutrients via mesoderm‐derived blood vessels during the transition from the area vitellina to the area vasculosa.^5^ In addition to a drastic morphological change of EECs during this transition,^6^ a differentiation process accompanied by the increase in expression of genes involved in nutrient uptake and lipoprotein metabolism, including the LRP2‐Cubilin‐Amnionless receptor tricomplex, Apolipoprotein B (*APOB*), *APOA1*, and *APOA5* occurs.^5^

In analogy to the EECs of the chicken yolk sac, the visceral endoderm of murine embryos comprises a single layer of polarized, columnar epithelial cells resting on the extraembryonic mesoderm, which is specialized for the efficient absorption and digestion of maternally derived nutrients.^1^, ^7^ Interestingly, most of the genes that we found to be up‐regulated during the vitellina‐to‐vasculosa transition in the chicken have previously been shown to be indispensable for mammalian embryonic development, while being exclusively expressed in the endodermal cells of the mammalian visceral yolk sac. For example, mice deficient in Cubilin (*CUBN*), Amnionless (*AMN*), *APOB*, or *MTP* die around day 10 of embryonic development (E10) with severe malformations such as neural tube defects and exencephaly.^8^, ^9^, ^10^, ^11^

Whereas the functions of these proteins in the EECs of the yolk sac are rather well established, the molecular mechanisms that underlie the differentiation and specification of EECs leading to expression of the respective genes are rather poorly understood. From the data obtained in our previous study,^5^ we concluded that signals from the mesoderm‐derived vasculature most probably induce differentiation of yolk sac EECs responsible for the observed differences in gene expression profiles in the developing chicken yolk sac.^12^ However, molecular signals dictating the transformation process of the EECs from a resting and lipid‐storing to a metabolically highly active phenotype have not been identified to date in the chicken system.

Bone morphogenetic proteins (BMPs) comprise a subgroup of the large family of transforming growth factor beta (*TGFB*) gene superfamily, and were originally identified by their capacity to induce cellular processes required for bone formation and differentiation.^13^ BMPs are secreted proteins and signal through type I and type II TGFB superfamily receptors. Upon ligand binding, the receptors form heterodimers (or heteromultimers) and trigger a downstream phosphorylation cascade of SMAD proteins. In vertebrates, seven type I receptors and five type II receptors are known, but only three type I receptors (BMP type I A receptor, BMP type I B receptor and the activin receptor like kinase, ACVRI or ALK2) and three type II receptors (BMPRII, ActRIIA, and ActRIIB) function as receptors for BMPs. Importantly, activated BMP‐bound type I receptor kinases phosphorylate SMAD1, 5, and 8, which heterodimerize with SMAD4 and translocate to the nucleus to affect gene expression (reviewed in Reference 13).

During murine yolk sac development, BMPs are primarily expressed in mesodermal angioblasts that eventually form the vascular network mediating nutrient transport to the embryo.^14^, ^15^ BMP4‐knockout mice die between E6.5 and E9.5, with embryos showing severe developmental retardation, disorganized posterior structures, and a reduction in extraembryonic mesoderm, including yolk sac blood islands.^16^ Since (a), targeted disruption of the BMP4 receptor ALK2 causes embryonic lethality, abnormal visceral endoderm morphology and disruption of mesoderm formation^17^ and (b), murine blastocyst‐derived extraembryonic endoderm (XEN) cells acquire features of endodermal cells of the yolk sac upon stimulation with BMP4,^18^, ^19^ our major goal was the elucidation of the role of BMP signaling in yolk sac endoderm specification. As the developing chicken yolk sac represents a valuable and amenable tool to study these developmental processes in detail, we set out to shed further light on the potential of BMPs in regulating chicken yolk sac function, and in particular, EEC differentiation.

## 2 RESULTS

**Figure 1:**
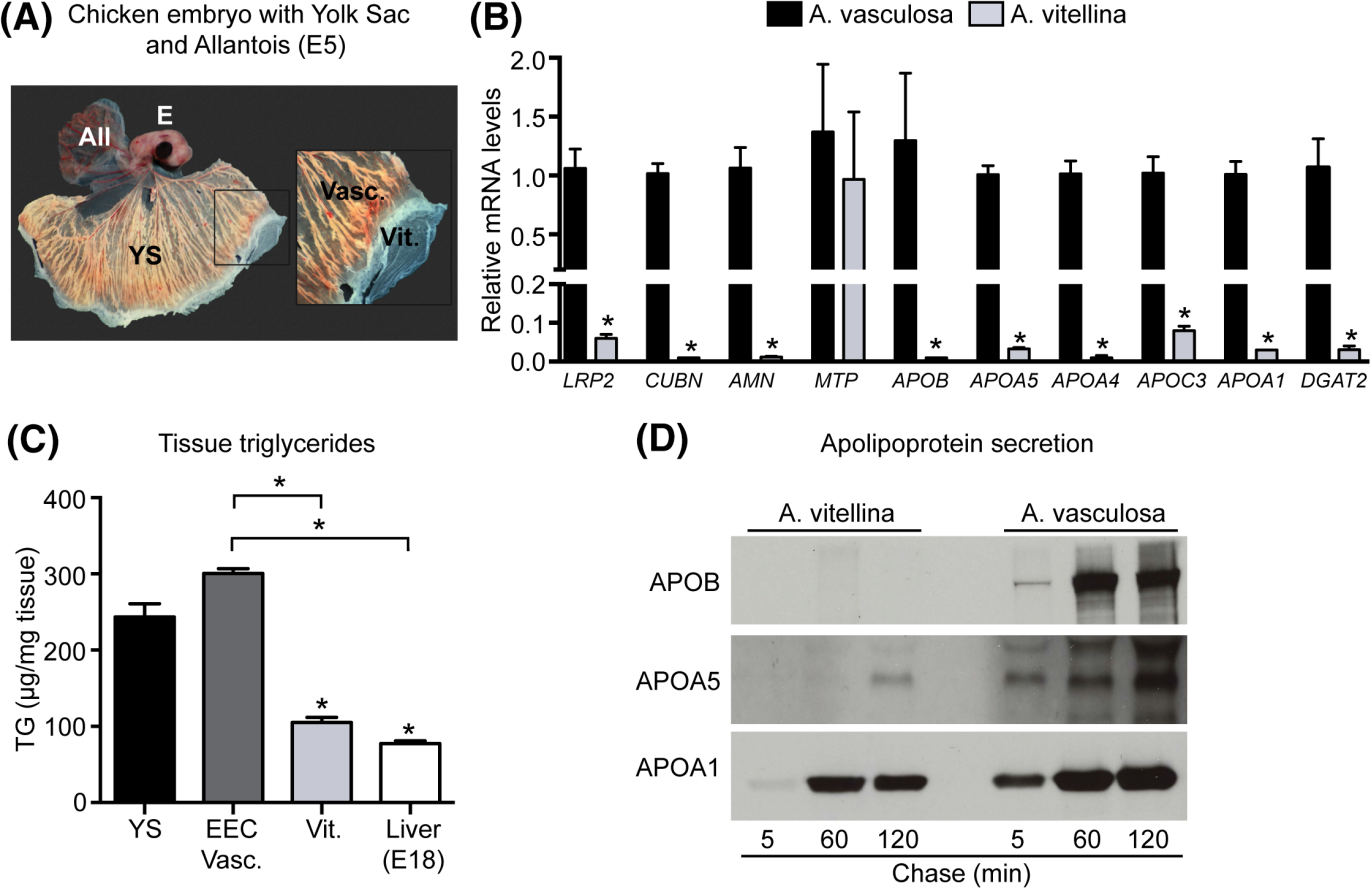
The transition from the area vitellina to the area vasculosa is accompanied by major changes in EEC gene expression, triglyceride content and apolipoprotein secretion. A, Chicken embryo (E) at day 5 of embryonic development (E5) with allantois (All, upper left) and yolk sac (YS) underneath. EECs of the area vitellina are located at the leading edge (Vit) preceding the vascularized, fully functional area vasculosa of the yolk sac (Vasc). B, Transcript levels of the indicated genes were analyzed with qPCR in isolated EECs from the area vasculosa (black bars) and the area vitellina (light grey bars). Relative gene expression levels were normalized to chicken *β‐actin* C, Quantification of triglyceride levels in tissue samples of total yolk sac from the area vasculosa (YS), isolated EECs from the area vasculosa (EECs Vasc.), EECs from the area vitellina (Vit.) and embryonic liver from E18 chicken embryos. D, Supernatants of radiolabeled EECs from the area vitellina and the area vasculosa were analyzed on their capacity to secrete apolipoproteins by immunoprecipiation. Results in B and C are presented as mean values ± SEM (n = 3 biological replicates) and statistical significance was calculated using Student's *t*‐test (*P < .05)

In our previous study,^5^ we showed that during the vascularization of the EECs, the yolk sac acquires the molecular machinery required for efficient uptake of yolk components, resynthesis of lipoproteins, and their (re)secretion into the embryonic circulation. To gain a more comprehensive understanding of the changes in lipid metabolism during the vascularization of the EECs (i.e., the area vitellina—to vasculosa transition, Figure 1A), we performed qPCR experiments with EECs from the area vasculosa and the area vitellina (Figure 1B). We found that in addition to the LRP2‐Cubilin‐Amnionless receptor tricomplex, which is expressed on the apical aspect of the EECs and mediates efficient uptake of yolk macromolecules,^5^ genes encoding apolipoproteins associated with triglyceride‐rich lipoproteins (*APOB*, *APOA5*, *APOA4*, and *APOC3*) as well as with HDL‐like particles (*APOA1*) are predominantly expressed in the EECs of the area vasculosa (Figure 1B). Additionally, *DGAT2*, the rate limiting enzyme in triglyceride synthesis and an essential component in the assembly and secretion of TG‐rich lipoproteins,^20^, ^21^ shows similar expression patterns as observed for the receptor tricomplex and apolipoproteins (Figure 1B). Whereas mRNA levels for the large subunit of microsomal triglyceride transfer protein (MTP), which mediates the lipidation of APOB essential for VLDL and chylomicron assembly,^22^ are comparable in the area vasculosa and vitellina (Figure 1B), MTP protein was exclusively detectable in the area vasculosa.^5^

Assembly and secretion of TG‐rich lipoproteins such as VLDL or chylomicrons requires not only the protein components, but also considerable amounts of triglycerides, which represent VLDLs' and chylomicrons' most abundant lipid species (reviewed in References 23, 24). Importantly, measurement of TG levels revealed a significant, three‐fold excess of TGs in the area vasculosa compared to the area vitellina EECs (Figure 1C). Accordingly, pulse chase experiments with EEC explant cultures revealed that APOB and APOA5, two apolipoproteins almost exclusively present on TG‐rich lipoproteins, were efficiently secreted from EECs of the area vasculosa, whereas APOA1 (in general a component of triglyceride‐poor lipoproteins) was secreted from EECs of both areas (Figure 1D). Together, these data are compatible with TG‐rich lipoprotein assembly and secretion in EECs of the area vasculosa, initiated by the progressive vascularization of EECs by mesodermally derived blood vessels.

**Figure 2:**
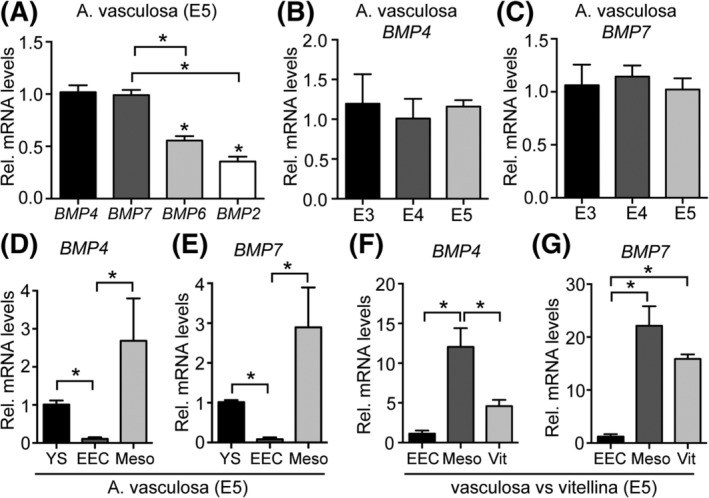
*BMP4* and *BMP7* are predominantly expressed in the ecto‐ and mesodermal cell layer of the chicken yolk sac's area vasculosa. A, Relative gene expression of chicken *BMP4*, *BMP7*, *BMP6*, and *BMP2* was measured with qPCR in E5 yolk sac area vasculosa tissue and normalized to chicken *β‐actin*. B, C, Relative gene expression of *BMP4* and *BMP7*, respectively, in the yolk sac's area vasculosa of chicken embryos from day 3 (E3) to day 5 (E5) of embryonic development. D, E, Relative gene expression of *BMP4* and *BMP7* was determined in dissected layers of the yolk sac's area vasculosa, normalized to chicken *β‐actin* and compared to the expression levels of the genes in the undissected area vasculosa of the yolk sac. F, G, Relative gene expression of *BMP4* and *BMP7* in EECs and mesodermal cell layer of the vasculosa compared to the expression levels in the area vitellina. Results are presented as mean values ± SEM (n = 3 biological replicates) and statistical significance was calculated using Student's *t*‐test (*P < .05)

Next, we aimed at the identification of a possible molecular mechanism orchestrating the observed differentiation of EECs. In studies with murine embryos, bone morphogenetic protein 4 (BMP4) has been identified as a candidate inducer of a mesenchymal‐to‐epithelial transition of extraembryonic endodermal cells, eventually resembling extraembryonic visceral endoderm (exVE), a subtype of visceral endoderm forming the definitive yolk sac in mammals.^18^, ^19^ To investigate whether BMP family members known to mediate developmental processes could also regulate EEC differentiation in the chicken yolk sac, mRNA levels of *BMP4*, *BMP7*, *BMP6*, and *BMP2* were measured using qPCR analysis of embryonic day 5 (E5) yolk sac tissue samples from the area vasculosa (Figure 2A). Interestingly, mRNA transcripts for *BMP4* and *BMP7* were present in comparable amounts, whereas expression levels of *BMP6* and *BMP2* were significantly lower (Figure 2A). Robust levels of *BMP4* and *BMP7* transcripts were readily detectable in the area vasculosa of E3 chicken embryos without time‐dependent regulation up to day 5 of embryonic development (Figure 2B,C). By separating the area vasculosa tissue into EECs and the associated mesodermal/ectodermal cell layers, we could localize *BMP4* and *BMP7* expression to the ectodermal/mesodermal cell sheet of the area vasculosa, whereas the endodermal cells hardly produced any *BMP* transcripts (Figure 2D,E). When further comparing *BMP* transcription levels between ecto/mesodermal cells of the area vasculosa and those of the area vitellina, *BMP4* was expressed at significantly higher levels in the area vasculosa (Figure 2F). Although a trend towards higher expression in the area vasculosa was also evident for *BMP7*, differences were not statistically significant (Figure 2G).

**Figure 3:**
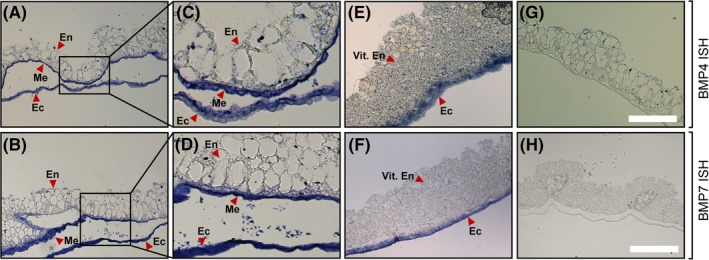
In situ hybridization of *BMP4* and *BMP7* in the area vasculosa and the area vitellina of yolk sac tissue at day 5 of embryonic development. A, B, Yolk sac area vasculosa tissue samples were subjected to in situ hybridization experiments with digoxigenin‐labeled probes targeting mRNA transcripts of chicken *BMP4* and *BMP7* (antisense) or G, H, nontargeting control (sense) probes. C, D, Magnifications of the inserts shown in A and B. E, F, Yolk sac area vitellina tissue samples were subjected to in situ hybridization experiments with digoxigenin‐labeled probes targeting mRNA transcripts of chicken *BMP4* and *BMP7* (antisense). En = Endodermal epithelial cells; Me = Mesodermal cell sheet; Ec = Ectoderm. Samples were embedded in paraffin and sections of 4 μm were prepared. Images were acquired using a Zeiss Axioscope. Magnification in A, B, E, F, G, H: 20×; scale bar, 200 μm

To morphologically gain a more comprehensive understanding of the localization of *BMP* expression, whole‐mount in‐situ hybridization experiments with E5 yolk sac tissue samples from the area vasculosa and the area vitellina were performed (Figure 3). Clearly, using specific *BMP4* and *BMP7* antisense oligonucleotide probes, both transcripts could be localized to the ectodermal and mesodermal cell layers that are in direct contact with the basolateral aspect of the EECs in the area vasculosa of the chicken yolk sac (Figure 3A,B). Higher magnifications more precisely illustrate *BMP* expression in both, ectodermal and mesodermal portions of the yolk sac and also show the tight association of BMP‐positive mesodermal structures with the endodermal absorptive epithelium (Figure 3C,D). Recapitulating our findings from qPCR analysis, the area vitellina ectoderm stained also positive for *BMP4* and *7* transcripts, albeit to a lesser extent compared to the area vasculosa (Figure 3E,F). Nonannealing sense RNA probes served as negative control (Figure 3G,H). These findings identified BMP4 and BMP7 as putative paracrine signals secreted from the ecto/mesodermal cell layer that might initiate the cellular differentiation of EECs during the area vitellina‐to‐vasculosa transition.

**Figure 4:**
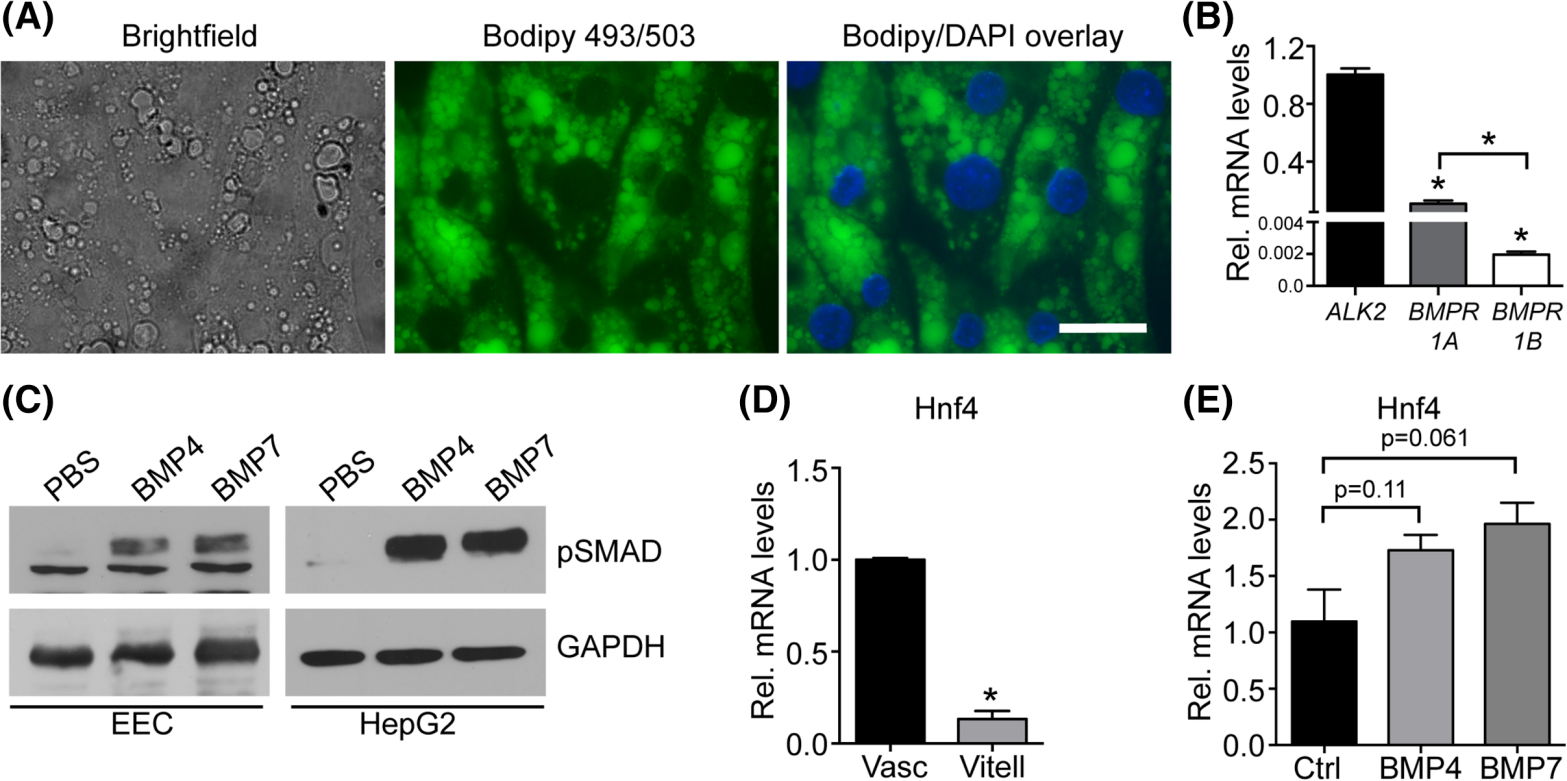
BMP signaling in the chicken yolk sac EECs is mediated through the BMP receptor ALK2 and its downstream mediator SMAD5. A, Light microscopic analysis of cultured EECs from the area vitellina. Granules observed in the brightfield view stain positive for the neutral lipid dye Bodipy 493/503. B, Relative gene expression levels of the potential type I BMP receptors *ALK2*, *BMPR1a*, and *BMPR1b* in EECs of the area vasculosa. C, Cultured EECs from the area vitellina and HepG2 cells were starved for 2 hours and subsequently stimulated with BMP4 or 7 for 40 minutes (200 ng/mL). SMAD5 phosphorylation was analyzed by Western blotting. GAPDH was used as loading control. D, Comparison of *HNF4* mRNA levels in EECs of the area vasculosa and area vitellina. E, qPCR analysis of *HNF4* mRNA levels in cultured EECs treated with either control PBS or recombinant BMP4 and BMP7. B, D, E, Relative gene expression levels are normalized to chicken *β‐actin* and represent the average of triplicate measurements. Results in B, D, and E are presented as mean values ± SEM (n = 3 biological replicates) and statistical significance was calculated using Student's *t*‐test (*P < .05)

BMPs bind to cell surface receptors of type I and type II serine/threonine kinases, which upon heterotetramerization induce SMAD‐dependent downstream signaling events leading to a diverse repertoire of cellular responses.^13^, ^17^ To directly demonstrate the capacity of BMPs to activate signaling pathways in EECs, we established a protocol to culture primary EECs in vitro. Due to the high concentration of intracellular triglycerides, EECs from the area vasculosa did not adhere to the cell culture dish. In contrast, explants of EECs from the area vitellina adhered well, and formed a confluent monolayer rich in intracellular granules and Bodipy‐positive lipid droplets after 2 days of culture (Figure 4A). qPCR analysis revealed that from the three type I serine/threonine kinases known to bind BMPs, type I activin receptor (ALK2) was expressed at highest levels in cultured EECs. In contrast, BMPR1A and BMPR1B showed only basal expression levels (Figure 4B). Importantly, treatment of EECs with either BMP4 or BMP7 induced phosphorylation of SMAD5 (Figure 4C, left panel), one of the central receptor‐regulated R‐SMAD proteins that act as transcription factors for a broad variety of target genes upon BMP‐receptor interaction.^18^, ^19^, ^25^, ^26^ As a control, treatment of the hepatocellular carcinoma cell line HepG2 resulted in comparable BMP‐mediated SMAD5 phosphorylation, confirming the results obtained with the primary EEC cultures (Figure 4C, right panel).

Although the secretion of APOB‐containing TG‐rich lipoproteins is mainly regulated via posttranslational mechanisms that control APOB lipidation and intracellular trafficking,^23^, ^24^ SMAD transcription factors have been demonstrated to directly bind to enhancer elements located 54 to 63 kb upstream of the *APOB* promoter region, thereby increasing *APOB* mRNA levels and protein secretion in intestinal cells.^27^ Furthermore, the TGFβ‐SMAD signaling axis has been shown to induce *APOCIII* in synergy with hepatocyte nuclear factor 4 alpha (HNF4α). The transcription factor HNF4α itself is induced upon BMP4 treatment in murine XEN cells^18^ and was identified as an important regulator of many genes involved in lipid metabolism, including *APOB* and *MTP*.^18^, ^19^, ^28^ Here, quantitative mRNA expression analysis revealed a robust increase in HNF4α mRNA levels in EECs of the area vasculosa compared to EECs of the area vitellina (Figure 4D), concurrent with the observed increase in the secretion of APOB (Figure 1D) and of VLDL^5^ in the area vasculosa of the yolk sac. Notably, BMP4‐ as well as BMP7‐treatment resulted in a trend towards increased mRNA levels of HNF4α in cultured EECs (Figure 4E), pointing to a BMP‐induced induction of HNF4α activity. Together, the responsiveness of EECs towards stimulation with BMPs in terms of SMAD5 phosphorylation as well as the induction of HNF4α expression might represent a synergistic signaling process that drives the induction of TG‐rich lipoprotein metabolism in the EECs of the area vasculosa.

**Figure 5:**
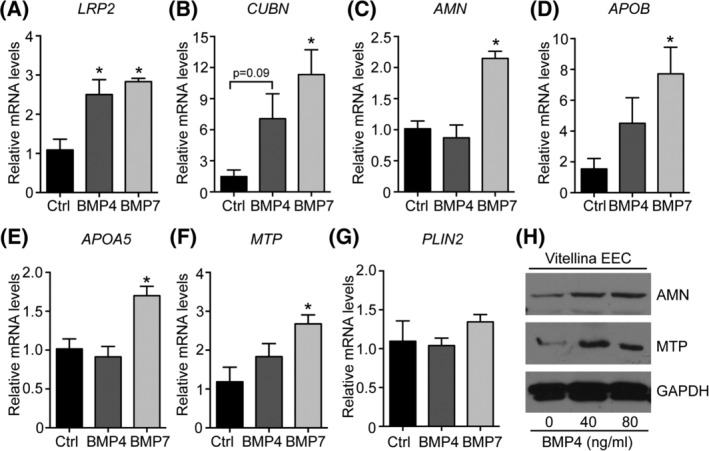
Stimulation of EECs with BMP4 and BMP7 induces an area vasculosa‐like gene expression pattern. A‐G, EECs from the area vitellina were cultured for 48 hours in the presence or absence of 200 ng/mL recombinant mouse BMP4 or BMP7. Relative gene expression levels were normalized to chicken *β‐actin* and compared to control treated EECs (n = 3, *P < .05, Student's *t*‐test). H, Cultured EECs from the area vitellina were treated with 0, 40, or 80 ng/mL recombinant mouse BMP4 and analyzed for the expression of AMN and MTP using Western blotting. GAPDH was used as loading control

**Figure 6:**
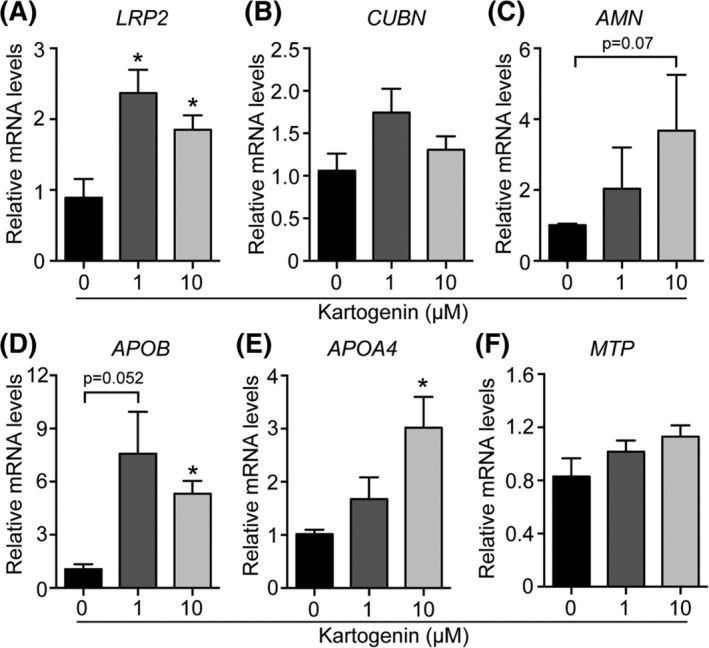
Kartogenin, a small molecule SMAD5 activator partially mimics BMP treatment. A‐F, EECs from the area vitellina were cultured for 48 hours in the presence of 0, 1, or 10 μM of the SMAD5 activator Karotgenin. Relative gene expression levels were normalized to chicken *β‐actin*. Results are presented as mean values ± SEM (n = 4 biological replicates) and statistical significance was calculated using Student's *t*‐test (*P < .05)

To further strengthen our hypothesis, we treated primary EECs from the area vitellina with either BMP4 or BMP7 and performed expression analysis of *LRP2*, *CUBN*, and *AMN*, encoding the endocytotic receptor tricomplex that transports nutrients from the yolk into the EECs. Likewise, we analyzed the expression of *APOB*, *APOA5*, and *MTP*, essential components of VLDL assembly and secretion. We observed that BMP4 treatment increased expression of *LRP2* and *CUBN* (*P* = .09, borderline significance), whereas *AMN* expression levels were unchanged (Figure 5A‐C). Although statistically not significant, BMP4 treatment increased the expression of *APOB* and *MTP* (Figure 5D,F). Interestingly, BMP7 treatment resulted in a more robust and significant increase in expression levels of the receptor tricomplex as well as of *APOB*, *APOA5*, and *MTP* (Figure 5A‐F). In contrast, neither BMP4 nor BMP7 had an impact on the expression of *PLIN2*, an area vitellina‐specific lipid droplet‐associated protein (Figure 5G).^5^ Finally, to confirm our results at the protein level, BMP4‐stimulated EECs were subjected to Western blot analysis. Unfortunately, it was not possible to detect LRP2, CUBN, APOB, or APOA5 protein in cultured EECs of the area vitellina due to limited amount of cellular material derived from the explant cultures and low expression levels of the respective proteins. However, and in partial contrast to results from qPCR analysis, BMP4 treatment resulted in an increase in AMN and MTP protein levels (Figure 5H). To further support our findings that BMPs induce a differentiation process in the endodermal cell layer, thereby promoting their nutrient transport competence, we performed experiments with explant cultures of endodermal cells from the area vitellina. To mimic BMPs action, we treated vitellina explants with the SMAD5 activator Kartogenin^29^, ^30^ and used expression patterns of genes of the receptor tricomplex and critical components of the TG‐rich lipoprotein assembly/secretion machinery as a surrogate marker for the nutrient transport competence of the EECs. Thereby, we found that Kartogenin treatment significantly induced *LRP2* expression (Figure 6A) and caused a trend towards increased expression of *CUBN* and *AMN* (Figure 6B,C). Importantly, the SMAD5 activator induced expression of *APOB* and *APOA‐IV*, two major structural components of TG‐rich lipoproteins such as VLDL and chylomicron particles (Figure 6D,E). In line with the results obtained for *MTP* in Figure 1B, we did not observe changes in *MTP* expression in area vitellina EEC explant cultures upon Kartogenin stimulation.

Altogether, the data appear to support a paracrine, BMP4/7‐mediated differentiation process of EECs that induces TG‐rich lipoprotein synthesis and secretion, providing the basis for efficient yolk‐sac‐mediated nutrient transport to the growing avian embryo.

## 3 DISCUSSION

**Figure 7:**
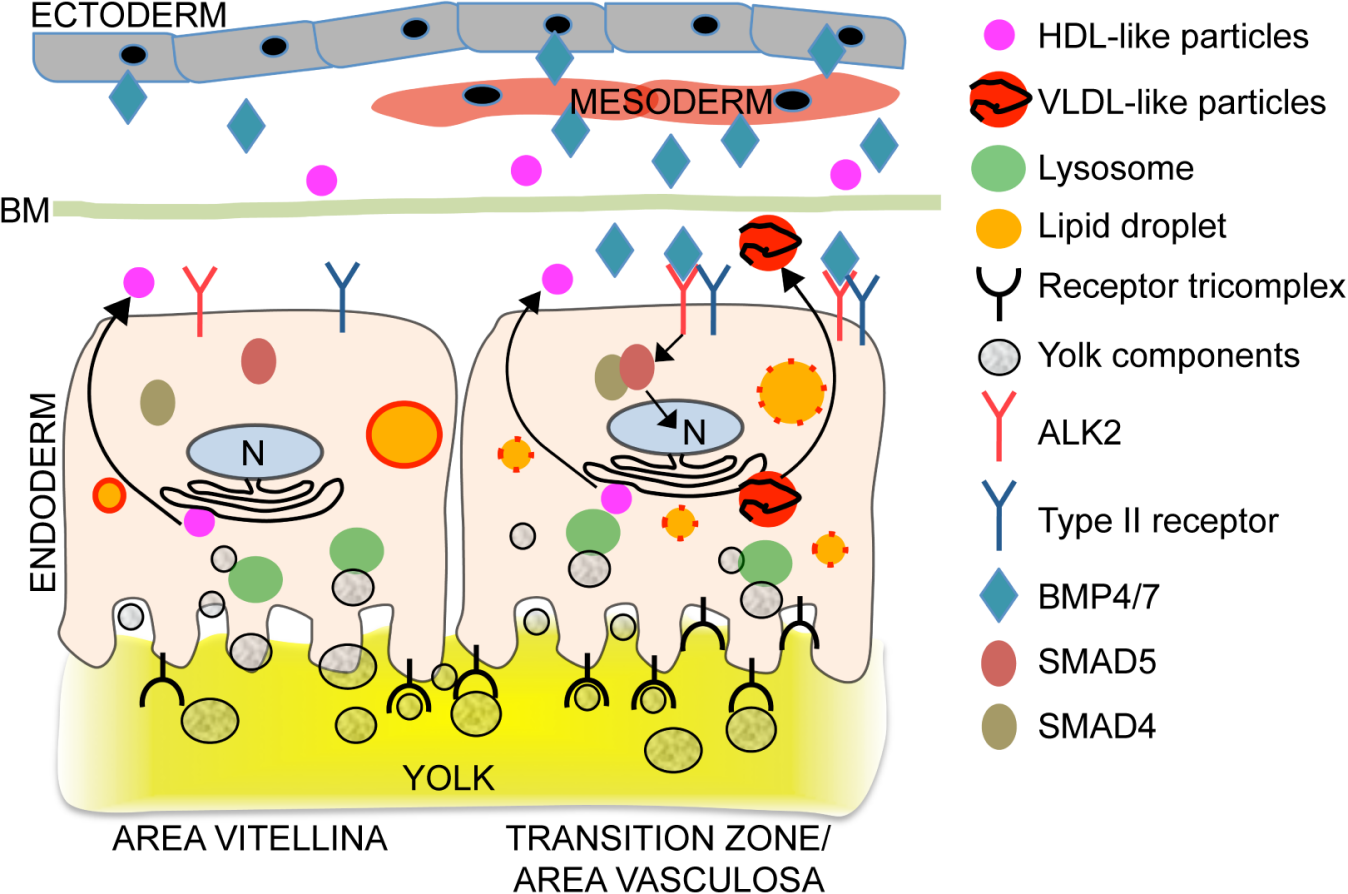
BMP signaling in the chicken yolk sac induces a differentiation process of EECs during the area vitellina to area vasculosa transition. In the absence of BMP signaling, EECs in the area vitellina (left‐hand side) remain in a resting state, characterized by the absence of APOB synthesis and secretion, the absence of the receptor tricomplex, and the expression of PLIN2. In the transition zone/area vasculosa (right‐hand side), BMP4 and BMP7, expressed and secreted from cells of ecto/mesodermal origin, are secreted into the extracellular space where they diffuse and eventually bind the type I receptor ALK2 on the surface of EECs. Activated ALK2 then phosphorylates SMAD5, which in turn translocates to the nucleus and initiates target gene expression and the differentiation process of EECs

The yolk sac endoderm provides the majority of lipid and protein components for the evolving chicken embryo, and supports early developmental processes in embryos of mammalian species.^1^, ^12^, ^31^, ^32^ As the growing chicken embryo relies exclusively on the yolk sac‐mediated supply with nutrients, the transformation of resting, lipid‐storing EECs in the area vitellina to metabolically highly active EECs in the area vasculosa represents a critical process during yolk sac maturation. Here we describe a control mechanism that coordinates the process of EEC‐differentiation during the progressive supply of EECs with mesodermally derived blood vessels. Together with data published in our previous study,^5^ we show that in the area vitellina, EECs in the absence of active BMP‐signaling secrete HDL‐like APOA‐I containing particles and contain PLIN2‐covered lipid droplets, but lack the competence to synthesize TG‐rich lipoproteins (Figure 7). During the vitellina‐to‐vasculosa transition, the invading mesodermal cell layer produces significant amounts of BMP4 and BMP7, which act in a paracrine manner on EECs, leading to SMAD5 phosphorylation and the induction of EEC differentiation and remodeling. In turn, the transcriptional profile of EECs changes dramatically so as to afford the efficient uptake of yolk nutrients as well as the synthesis and resecretion of potentially nutritive TG‐rich lipoproteins (Figure 7). Therefore, mesodermally derived BMPs likely represent crucial paracrine signaling components that ensure proper development and function of the yolk sac EECs.

Interestingly, extensive research with murine embryos identified signals from the yolk sac's endodermal cells essential for the mesodermal cell layer to develop vascular tubes and mature blood vessels.^15^ Indian‐ and sonic hedgehog proteins secreted from the endodermal cell layer of the yolk sac signal to the invading, vasculogenesis‐competent mesodermal cells, thereby initiating FOXF1 expression. Of note, FOXF1‐deficient mouse embryos show markedly decreased BMP4 expression in the mesoderm of the yolk sac, an essentially avascular yolk sac phenotype, and early resorption of the fetus. Importantly, the exogenous addition of BMP4 was sufficient to induce vascular tube‐ and blood vessel formation in the yolk sac.^33^, ^34^ Thus, BMPs are key to normal mesodermal vasculogenesis that is a result of the crosstalk between the closely associated mesodermal and endodermal cell layers during the formation of the yolk sac.

In contrast, detailed studies on the role of BMPs in the differentiation and specialization program of endodermal yolk sac cells are rather scarce, likely due to the limited amount and poor accessibility to tissues. However, the isolation of stem cell lineages from murine blastocysts, among them the parietal endoderm‐derived extraembryonic endoderm (XEN) cell line, has provided a valuable tool to study this intercellular crosstalk.^18^, ^35^ Stimulation of XEN cells with BMP4, expressed in the visceral endoderms' adjacent extraembryonic ectoderm and mesoderm during mouse embryogenesis, established an apical‐basal polarity. In addition, expression profiling of these cells showed that genes normally expressed in the visceral endoderm of the yolk sac, including *Lrp2*, *Cub*, and *Amn*, as well as genes encoding apolipoproteins such as *ApoA‐I*, *ApoA‐2*, *ApoE*, and *ApoM* were significantly up‐regulated.^18^, ^19^ Interestingly, BMP4‐treated XEN cells show increased expression of vascular endothelial growth factor and Indian hedgehog, which previously have been implicated in primitive hematopoiesis and vasculogenesis^18^ by activating BMP4 expression in mesodermal angioblasts in the yolk sac.^33^ These findings complement our current data and suggest a paracrine crosstalk that orchestrates the differentiation and specification of the yolk sac's EECs as well as the mesodermally derived vasculature. Our results from qPCR and in situ hybridization experiments revealed that BMPs are readily detectable in area vitellina ectodermal cells in proximity to the sinus terminalis of the area vasculosa (Figures 2F,G and 3E,F). From a functional perspective, the BMP signal in the area vitellina might also be involved in the structural rearrangement of the endodermal cells from a multilayered cell sheet towards a polarized, highly organized epithelium in the area vasculosa.^36^ Interestingly, Kartogenin, which induced a vasculosa‐like gene expression pattern in EECs from the area vitellina (Figure 6), was previously shown to promote collagen synthesis in human dermal fibroblasts.^30^ Of note, collagen is a well‐known component of basement membranes and the extracellular matrix, two important factors that regulate epithelial polarization.^37^

Stimulation of cultured chicken EECs from the area vitellina with either BMP4 or BMP7 resulted in a robust increase in expression of the receptor triad LRP2, CUBN and AMN, which are well known to act as a multiligand receptor complex with high endocytic capacity in the small intestine, the proximal tubules of the kidney, and the endodermal cells of the yolk sac.^38^, ^39^, ^40^ That the receptor tricomplex plays a crucial role in proper nutrient supply during embryonic development has been demonstrated in several studies.^11^, ^39^, ^40^, ^41^ Cubilin, for example, was identified as the target of teratogenic antibodies when injected into pregnant rats, as they localized almost exclusively to clathrin‐coated pit structures of the visceral endodermal cells of the yolk sac.^42^ Subsequent studies in mice showed that the lack of either *LRP2*, *CUBN*, or *AMN* leads to similar embryonic‐lethal phenotypes with defects in gastrulation, mesoderm formation, and neural tube closure.^9^, ^11^, ^41^ In humans, neural tube closure usually occurs at very early stages of development, that is, before the establishment of the chorioallantoic placenta.^3^ In this critical time window, nutrients are absorbed by histotrophic mechanisms involving phagocytosis as well as receptor‐mediated endocytosis. Thereby, despite structural differences, the murine and most likely also the human yolk sac's visceral endodermal cells function as a fetomaternal interface that mediates absorption and transport of nutrients to the growing embryo.^3^, ^4^, ^39^ Additional evidence for an important role of LRP2/CUBN/AMN‐mediated transport of nutrients to the early embryo is provided by studies that analyzed the composition of the coelomic fluid, which is in direct contact with the endodermal cells of the visceral yolk sac and is rich in vitamin A, B12, D, cobalamine, and cholesterol. These macromolecules are ligands for the receptor tricomplex, which binds, internalizes, and thereby provides the accessibility of these factors to the growing embryo.^3^, ^38^, ^40^

Our current data indicate that BMPs activate SMAD signaling and induce the transcription factor HNF4α in EECs of the chicken embryo, apparently resulting in a synergistic target‐gene activation. Notably, *ALK2*‐deficient mice show reduced expression of the visceral endoderm‐specific transcription factor HNF4α,^25^, ^43^ which is highly expressed in the liver, small intestine, and kidney, as well as in the endodermal cells of the yolk sac.^28^, ^44^, ^45^ HNF4α was recently shown to activate LRP2 expression in proximal tubule cells of the kidney by directly binding to an HNF4α‐binding site in the LRP2 promoter.^45^ The critical importance of HNF4α in embryonic development was first recognized in studies in *Drosophila*, where maternal HNF4α mRNAs are present in the fertilized egg, deposited by nurse cells. In later stages, HNF4α mRNA is detected in endodermal cells of the midgut primordium, showing some overlapping expression pattern with that found in rodents, in which HNF4α expression is restricted to the cells of the visceral endoderm, starting before gastrulation.^44^, ^46^ HNF4α‐deficient mice die early during gestation with severe malformations including gastrulation defects, absence of mesodermal structures, and growth retardation compared to wild‐type and heterozygous littermates.^47^ However, it is not completely clear whether embryonic lethality is due to decreased absorptive function of the yolk sac.^47^

The vascularization of the chicken yolk sac's EECs induces a massive turnover of yolk‐derived TGs and the secretion of TG‐rich, APOB containing VLDL‐like particles (Figure 1 and^5^). Supporting evidence for our findings that the BMP‐SMAD‐HNF4α signaling axis might also regulate APOB expression and VLDL secretion in the developing yolk sac is the identification of binding sites for HNF4α, HNF3β and SMAD transcription factors in the *APOB* promoter. Of particular interest is the finding that SMADs regulate *APOB* transcription in a tissue‐specific manner. By binding to an enhancer element 55 kb upstream of the APOB transcription start, SMAD proteins increase *APOB* transcription in human intestinally derived Caco2 cells, whereas they attenuate transcription in HepG2 cells.^27^ Of note, the small molecule SMAD5 activator Kartogenin significantly induced mRNA levels for *APOB* and *APOA4* in explant cultures of EECs from the area vitellina (Figure 6D,E). These experiments provide further evidence for an important contribution of SMAD signaling in the process of TG‐rich lipoprotein assembly and secretion in the yolk sac, an absorptive epithelium with unequivocal structural similarities to the small intestine. Finally, liver‐specific ablation of HNF4α in adult mice results in the development of fatty liver and reduction of plasma TG levels by 80%, a consequence of reduced VLDL secretion, whereas overexpression of HNF4α significantly induced mRNA levels of APOB and MTP.^28^

Besides the differential transcriptional regulation of key components of TG‐rich lipoproteins in the area vasculosa vs. the area vitellina, a block to production of TG‐rich particles might be the presence of the lipid droplet‐stabilizing protein PLIN2 in the EECs of the area vitellina.^5^ PLIN2 was previously shown to hamper VLDL lipidation by protecting the fatty acid pool stored in lipid droplets from lipolysis, thereby decreasing TG incorporation into VLDL particles.^48^ Furthermore, PLIN2‐knockout mice showed an increased VLDL secretion rate and a reduction in hepatic TG content.^49^

In summary, our study (a) provides molecular evidence for a regulatory crosstalk between the cell layers that form and functionally establish the avian yolk sac and (b) sheds light on paracrine signaling mechanisms that dictate the differentiation of EECs. Given the highly conserved structure and the critical importance of the mammalian yolk sac in early gestational periods, that is, neural tube closure and somite formation, the chicken embryo provides an excellent model for the analysis of yolk sac‐dependent developmental defects.

## 4 EXPERIMENTAL PROCEDURES

### 4.1 Animals

Sexually mature Derco brown (TETRA‐SL) laying hens were purchased from Diglas Co. (Feuersbrunn, Austria) and maintained as described in.^5^ Fertilized eggs were incubated in a humidified chamber at 38°C to maintain normal embryonic development.

### 4.2 Antibodies

The following in‐house raised rabbit polyclonal antisera/antibodies were used: anti‐ggApoA‐I antiserum^50^; anti‐ggApoA‐V antiserum^51^; and anti‐ggAPOB antibody.^50^ Rabbit monoclonal anti‐human SMAD5 (phospho S463 + S465, ab92698) and mouse monoclonal anti‐rabbit GAPDH (G8795) antibodies were purchased from Abcam and Sigma‐Aldrich, respectively.

*Preparation and dissection of yolk sac layers* was performed as described precisely in Reference 5. Yolk sac tissues used for all experiments in this study were obtained from embryos at day 5 of embryonic development.

### 4.3 Quantitative PCR

Total RNA was isolated from chicken tissues or from cultured cells using TRI reagent (MRC), and cDNAs were prepared by rev‐transcription of 1 μg of RNA using SuperScriptII rev transcriptase (Invitrogen) and oligo(dT) primers. cDNAs were subjected to quantitative PCR (qPCR) experiments essentially as described in Reference 5. The primers used are listed in Table 1. mRNA levels of genes are expressed as fold change to a respective control and represent the average of triplicate measurements ± SEM. Chicken β‐Actin was used as a housekeeping gene.

**Table 1:** Oligonucleotide primers and probes used for qPCR analysis and in situ hybridization, respectively

qPCR primers	Sequence (5′‐3′)	Amplicon size (bp)	Accession number
*ggApoA‐I* fwd	GACCGCATTCGGGATATGGT	197	NM_205525
*ggApoA‐I* rev	ATCTCGCGCACCTCCTTGTA		
*ggApoA‐IV* fwd	ATCACCAAGCAGCTCAACACC	201	NM_204938
*ggApoA‐IV* rev	CACCTCGTCGGCGTAAGGA		
*ggApoC‐III* fwd	AAAGTGGGAACTCGGGGCAG	251	XM_025143168
*ggApoC‐III* rev	GCCATTTCCTGGCTTGCTGAG		
*ggApoA‐V* fwd	CGCTGATAAGATCGCCTTCC	213	XM_025143219
*ggApoA‐V* rev	TGGATGTTGCGGTGCAGATC		
*ggApoB* fwd	CTCCATCAGAGCAGTCGAGT	216	NM_001044633
*ggApoB* rev	ATTAGGCCTCAGGGACAGTG		
*ggMtp* fwd	GCAGACGCCAGCATCTCT	256	NM_001109784
*ggMtp* rev	TACAACAGCCGGAAGCTTGC		
*ggβ‐Actin* fwd	AGCTATGAACTCCCTGATGG	236	NM_205518
*ggβ‐Actin* rev	ATCTCCTTCTGCATCCTGTC		
*ggLrp2* fwd	GGAGTGTTAGCGGTTGGAGGC	349	XM_004942763
*ggLrp2* rev	CCACACTACCAGCTCCTGTTA		
*ggCubilin* fwd	TGAACTCTCTGGATGGCTTC	264	XM_025148394
*ggCubilin* rev	CTCGTTCTGCATCAACACAG		
*ggAmn* fwd	TCTGGCTCTGGGTTCACAGC	268	NM_001277516
*ggAmn* rev	GAACAGGGATCACTCGCCG		
*ggPlin2* fwd	CCCACTGACAAGGTTGTTGCC	211	NM_001031420
*ggPlin2* rev	GCACCACACGACTTCCCAAG		
*ggAbca1* fwd	GCACTGAAGATGATGTAACT	205	NM_204145
*ggAbca1* rev	AGCTCCTGGAATGTCCTGTTCAC		
*ggDgat2* fwd	GGTGAAAACCCACAACCTGC	218	XM_419374
*ggDgat2* rev	TCACGGGACATATACCCCCA		
*ggHnf4α* fwd	AAGCAGCCAACCTCAACACC	248	XM_015296366
*ggHnf4α* rev	CTTCATCCCTGCTCGGAAGC		
*ggBmp2* fwd	GCAGCTTCCACCACGAAGAAGT	240	NM_204358
*ggBmp2* rev	TCGTGACAGGGTCCTTGGAG		
*ggBmp4* fwd	AGCTTCCACCATGAAGAGCACC	216	NM_205237
*ggBmp4* rev	CTCCGACAGCGGCTTCATCA		
*ggBmp6* fwd	CCAAGTGCTGCAGGAACATCC	188	XM_015275997
*ggBmp6* rev	TTACACTGAAACCGTCGTGGG		
*ggBmp7* fwd	GGAGCACCCAGGAAGGGATT	226	XM_417496
*ggBmp7* rev	CTGCTTGTTTTGTGGGCCGT		
*ggAlk2* fwd	CTATCTGCAGCTCACCACCC	227	NM_204560
*ggAlk2* rev	CCAACTGGTTCGTGCTTTGG		
*ggBmpr1a* fwd	ACCAACACTTGCTTCTGGCT	193	NM_205357
*ggBmpr1a* rev	GACTGCCATCCAACGAATGC		
*ggBmpr1b* fwd	ACTTCAGGTACAAGCGGCAA	216	NM_205132
*ggBmpr1b* rev	AGACTTCCCCATAGCGACCT		

*ISH oligonucleotides*	(5′‐3′); underlined: T7 RNA polymerase promoter sequence	Probe length (bp)	Accession number
anitsense *ggBMP4* fwd	AGCTTCCACCATGAAGAGCACC	724	NM_205237
antisense *ggBMP4* rev	TAATACGACTCACTATAGGGCGGA GTTGACCAACGTCTGC		
sense *ggBMP4* fwd	TAATACGACTCACTATAGGGAGCT TCCACCATGAAGAGCACC	724	NM_205237
sense *ggBmp4* rev	CGGAGTTGACCAACGTCTGC		
antisense *ggBMP7* fwd	GGAGCACCCAGGAAGGGATT	766	XM_417496
antisense *ggBMP7* rev	TAATACGACTCACTATAGGGTTCC CCTTCCCCACACATCC		
sense *ggBMP7* fwd	TAATACGACTCACTATAGGGGGA GCACCCAGGAAGGGATT	766	XM_417496
sense *ggBMP7* rev	TTCCCCTTCCCCACACATC		

### 4.4 Culture of endodermal epithelial cells from the area vitellina

The area vitellina of yolk sacs was dissected from the area vasculosa as previously described.^5^ The intact cell sheets of the area vitellina were disrupted by repeated pipetting until a homogenous cell suspension was obtained. Cells were resuspended in fresh DMEM‐F12 medium (Sigma) supplemented with 10% FCS, 2 mM l‐glutamine, 0.1 mg/mL streptomycin, and 100 units/mL penicillin, plated at high density on sterile 6‐well cell culture dishes and grown for 48 hours in complete DMEM‐F12 medium at 37°C and 5% CO_2_. For the detection of SMAD5 phosphorylation, EECs were washed 2× in PBS, starved for 2 hours in Opti‐MEM reduced serum medium followed by the incubation of the cells with recombinant mouse BMP4 or BMP7 proteins (R&D systems) at the indicated concentrations for 40 minutes. For the analysis of gene expression in BMP‐stimulated EECs, freshly isolated EECs were cultured for 48 hours in complete DMEM‐F12 medium containing 200 ng/mL recombinant BMP4 or BMP7 proteins as indicated in the figure legends (medium and BMPs were replaced after 24 hours).

### 4.5 Culture of human hepatocellular carcinoma (HepG2) cells

HepG2 cells were obtained from ATCC and cultured in MEM medium (PAA) supplemented with 10% FCS, 2 mM L‐glutamine, 0.1 mg/mL streptomycin, and 100 units/mL penicillin at 37°C and 5% CO_2_. Downstream experiments were exactly performed as described for EECs.

### 4.6 Preparation of protein lysates from cultured cells

After washing the cells in PBS, RIPA buffer (50 mM Tris/HCl, pH 7.4, 150 mM NaCl, 1% Nonidet P‐40, 1% sodium deoxycholate, 0.1% SDS, and complete proteinase and phosphatase inhibitor cocktails) was directly added to the dish, and the cells were detached using a cell scraper. For complete lysis, cells were pipetted up and down several times and sonicated for 10 seconds with constant pulse. After incubation of the lysates for 5 minutes at 4°C, samples were centrifuged for 45 minutes at 10.000 × *g* and 4°C. The obtained supernatants were immediately used for Western Blotting or stored at −80°C until use.

### 4.7 Western blotting

Western blotting was performed as described in Reference 5. Briefly, protein samples were analyzed by one‐dimensional SDS‐PAGE and electrophoretically transferred to nitrocellulose membranes (Hybond‐C Extra, AmershamBiosciences) using semidry blotting. Nonspecific binding sites were blocked with TBS (20 mM Tris‐HCl, pH 7.4, 140 mM NaCl) containing 5% (wt/vol) BSA and 0.1% Tween 20 (blocking buffer) for 1 hour at room temperature. Proteins of interest were detected with the indicated specific primary antisera/antibodies, followed by incubation with HRP‐conjugated secondary antibodies and development with the enhanced chemiluminescence protocol (Pierce).

### 4.8 Pulse‐chase radiolabeling experiments and immunoprecipitation

Five hundred milligrams EEC tissue fragments (either from the area vitellina or the area vasculosa) were washed extensively in PBS and subjected to pulse‐chase radiolabeling experiments as described in detail in Reference 5. Immunoprecipitates were subjected to SDS‐PAGE and subsequent autoradiography.

### 4.9 Extraction and quantification of triglycerides (TG)

Whole yolk sac tissue, EECs from either the area vasculosa or the area vitellina, or liver tissue from a mature laying hen (60‐80 mg each) were minced and transferred to glass vials containing 3 mL chloroform: methanol (2:1) and incubated for 15 minutes at 55°C followed by an incubation for 16 hours at room temperature. After centrifugation for 30 minutes at 500 × *g* at room temperature, the lipid fraction from each sample was collected and organic solvents were evaporated in a Bartelt HR‐1 Hetovac vacuum centrifuge. Lipids were resuspended in 1.5 mL chloroform: methanol (2:1), and samples were incubated for 5 minutes at 55°C. After the addition of 0.3 mL diluted H_2_SO_4_ (0.05%), the samples were vortexed and centrifuged at 500 × *g* for 15 minutes. Equal aliquotes (150 μL) of the lower lipid phase were mixed with 300 μL of 1% Triton X‐100 in chloroform and dried in the vacuum centrifuge. Pellets were resuspended in ultrapure H_2_O (150 μL), and TG concentration was measured using the Wako LabAssay Triglyceride Kit. Total TG concentrations were normalized to tissue wet weight.

### 4.10 In‐situ hybridization

Digoxigenin‐labeled in situ probes directed against regions of chicken *Bmp4* (NM_205237.3) and *Bmp7* (XM_417496.4) were generated by in vitro transcription of PCR products containing the T7 RNA polymerase promoter sequence at their 3′ (antisense probes) or 5′ (sense probes) ends.^5^ In situ hybridization analysis was carried out exactly as described at the GEISHA (Gallus expression in Situ Hybridization Analysis) Web site (“whole‐mount in situ hybridization protocol for mRNA detection”). This protocol represents a slight modification of those published.^52^, ^53^ After the staining procedure, tissue pieces were embedded in paraffin and sections of 4 μm were prepared. Slides were mounted with Fluorescence Mounting Medium (Dako) and images were acquired with a Zeiss Axioskope.

### 4.11 Statistics

Data represent mean ± SEM of representative experiments, unless otherwise stated. To compare the means of two groups, an unpaired, two‐tailed Student's *t*‐test was used. In all cases statistical significance was assumed when *P* < .05.
